# Real-World Case Series on Surgi-ORC® in ENT Surgery

**DOI:** 10.7759/cureus.95539

**Published:** 2025-10-27

**Authors:** Piyush Patel, Diksha Sharma, Bhavin Trivedi, Shahid Karatela, Abhinav Bhatnagar

**Affiliations:** 1 Quality, Aegis Lifesciences Pvt. Ltd, Ahmedabad, IND; 2 Research and Development, Medical Device Industry, Gujarat, IND; 3 Regulatory Affairs, Aegis Lifesciences Pvt. Ltd, Ahmedabad, IND; 4 Regulatory Affairs and Quality Assurance, Aegis Lifesciences Pvt. Ltd, Ahmedabad, IND; 5 Orthopedics, Abhinav Multispeciality Hospital, Nagpur, IND

**Keywords:** bleeding control, ent surgery, haemostasis, oxidized regenerated cellulose (orc), parotidectomy, surgi-orc®, thyroidectomy

## Abstract

Effective hemostasis is essential in ENT surgical operations to reduce blood loss and improve postoperative recovery. This case series assesses the hemostatic efficacy and safety of Surgi-ORC^®^ Knit, an absorbable oxidized regenerated cellulose (ORC) hemostat, in four patients undergoing four different ENT operations. This case series included four patients who underwent parotidectomy, thyroidectomy, neck mass excision, and submandibular excision. To control bleeding, a Surgi-ORC^®^ Knit was applied to the bleeding site. The study's primary objective was to monitor time to hemostasis for bleeding control in ENT surgery. The secondary aim of safety monitoring was to conduct a 60-day follow-up to confirm complete absorption and assess for adverse events. Along with this, the handling characteristics of the product were also assessed. Surgi-ORC^®^ attained hemostasis within the range of 0.93 to 2.1 minutes. No adverse events were reported, and all patients had stable recoveries. The surgeons found that the product was easy to apply and had good tissue adherence. Surgi-ORC^®^ was entirely absorbed within 28 days, which was confirmed by radiological imaging. On follow-up day 60, there were no signs of infections, adhesions, granuloma formation, or foreign body reactions. Surgi-ORC^®^ Knit is a viable treatment option for managing bleeding in ENT surgeries. Surgeons found its user-friendly application, stability, and easy delivery in intricate surgery locations to be noteworthy advantages. Its safety is bolstered by complete absorption and no further complications.

## Introduction

Bleeding is a significant concern in otorhinolaryngological (ENT) surgeries due to sensitive anatomy and highly vascularized structures, including blood vessels and nerves [1]. Different forms of ENT surgery present varied levels of hemorrhagic risk. Nasal endoscopic surgery, typically conducted for chronic rhinosinusitis unresponsive to conservative conventional treatments, identifies bleeding as the most prevalent minor or major consequence [2]. Postoperative bleeding ranges from 0.7% to 5% in endoscopic sinus surgery [3]. While posterior nasal neurotomy is considered a safe technique, it poses a high risk of bleeding compared to other endoscopic procedures. Endoscopic modified medial maxillectomy, an effective technique to reshape the maxillary sinus, has a higher risk of operative bleeding [4]. The throat, particularly the tonsillar region, has a vibrant blood supply, which leads to an increased risk of bleeding during tonsillectomy; it can result in hypovolemic shock, airway obstruction, or even death [5]. Due to the significant risks linked to numerous ENT operations and the possibility of severe consequences, efficient intraoperative hemostasis is essential for surgical success. However, even with improvements in surgical techniques, there is still a shortage of standardized, effective, and biocompatible topical hemostatic agents that have been specifically tested for use in otolaryngology. Traditional surgical methods, such as electrocautery and nasal packing, possess inherent limits. In otolaryngology, electrocautery is a commonly employed surgical instrument for managing hemorrhage; nonetheless, it presents several limitations and risks that must be considered for patient safety. Severe burns may occur due to the wrong placement of devices or the creation of a conductive pathway from the deposition of blood, sweat, and other physiological fluids. Incorrect patient placement may lead to skin folds, increasing the likelihood of decubitus ulcers that resemble low-impedance outlet burns [6]. This has resulted in escalated interest in supplementary topical therapies that are safe, are biocompatible, and facilitate fast hemostasis. Oxidized regenerated cellulose (ORC) is a biodegradable plant-derived hemostat that enhances clotting by offering a physical matrix to stimulate platelet aggregation [7]. However, the use of ORC is associated with certain disadvantages, including prolonged presence at the surgical site, risk of foreign body granuloma, and interference with postoperative imaging. Surgi-ORC® (Aegis Lifesciences Pvt. Ltd., Ahmedabad, Gujarat, India) is a new hemostatic agent made from ORC. It was created to fix the shortcomings of traditional ORC materials. It allows for quicker absorption, better flexibility, and easier handling. While its safety and effectiveness have been shown in orthopedic and general surgeries, there are limited data on its use in ear, nose, and throat surgeries. Therefore, it is important to assess its real-world performance in ENT procedures to confirm its suitability for this specific anatomical and clinical setting.

Uniform consistency and structural integrity of Surgi-ORC® improve hemostasis by enhancing clot formation. It maintains its mechanism of action in controlling localized bleeding. In a complex procedure involving intricate anatomical structures, bleeding control is engaged. To address this, Surgi-ORC® becomes a meticulously designed viable option, easily implanted at the surgical bleeding site with excellent adhesion to tissue. High tensile strength in wet and dry conditions allows for flexible use.

The safety and efficacy profile of Surgi-ORC® has already been established, which confirms its complete absorption within 7-14 days [8]. Surgi-ORC® Knit is three times denser, leading to >36% faster hemostasis. Its knitted design allows for suture retention, making it particularly advantageous in ENT procedures where precise bleeding control is crucial. The current case series was designed to provide real-world evidence on the safety, handling, and hemostatic effectiveness of Surgi-ORC® in ENT surgeries. This aims to fill the existing gap in data on this specialty.

## Case presentation

This was a case series of four patients who underwent ENT surgery at Abhinav Multispecialty Hospital, Nagpur, India, and Shri Shankaracharya Institute of Medical Sciences, Chhattisgarh, India. In clinical environments, Surgi-ORC® Knit was used to assess its effectiveness and safety in attaining fast hemostasis during diverse orthopedic surgical interventions. Patients were included if they were scheduled for ENT procedures associated with a moderate-to-high risk of intraoperative bleeding (e.g., nasal endoscopic surgery, tonsillectomy, endoscopic medial maxillectomy) and provided written informed consent. Exclusion criteria included known hypersensitivity to ORC, coagulation disorders, ongoing anticoagulant therapy, or systemic conditions likely to interfere with hemostasis. Surgi-ORC® Knit, a novel ORC-based hemostatic agent, was applied to bleeding sites during surgery. The product is supplied in sterile, single-use packaging designed for easy handling and minimal contamination. To ensure consistent use, the surgical team received training, including an Instructions for Use (IFU) guide, outlining product application, resizing, layer separation, placement on the bleeding site, and safe disposal of excess material. The primary outcome of the study was time to hemostasis (TTH), measured from the application of Surgi-ORC® Knit to the cessation of active bleeding. TTH was measured using a calibrated stopwatch, starting from the moment of Surgi-ORC® application to complete cessation of visible bleeding. The surgeon questionnaire used to collect feedback on product usability aspects was derived from the EC-approved PMCF protocol and Case Report Form (CRF). Measurements were recorded by the operating surgeon or a designated team member. Secondary outcomes included the ease of handling and application, as reported by the operating surgeons; the absorption of Surgi-ORC® Knit at the surgical site assessed clinically and, where feasible, through imaging; and safety, evaluated by monitoring for adverse events or complications over a 60-day follow-up period. The study was approved by the Jasleen Hospital Ethics Committee, Nagpur, India, and is registered with the Clinical Trials Registry of India (CTRI/2024/02/062445-ENT/003). All procedures adhered to ethical standards and ICH-GCP guidelines.

Case 1

**Figure 1:**
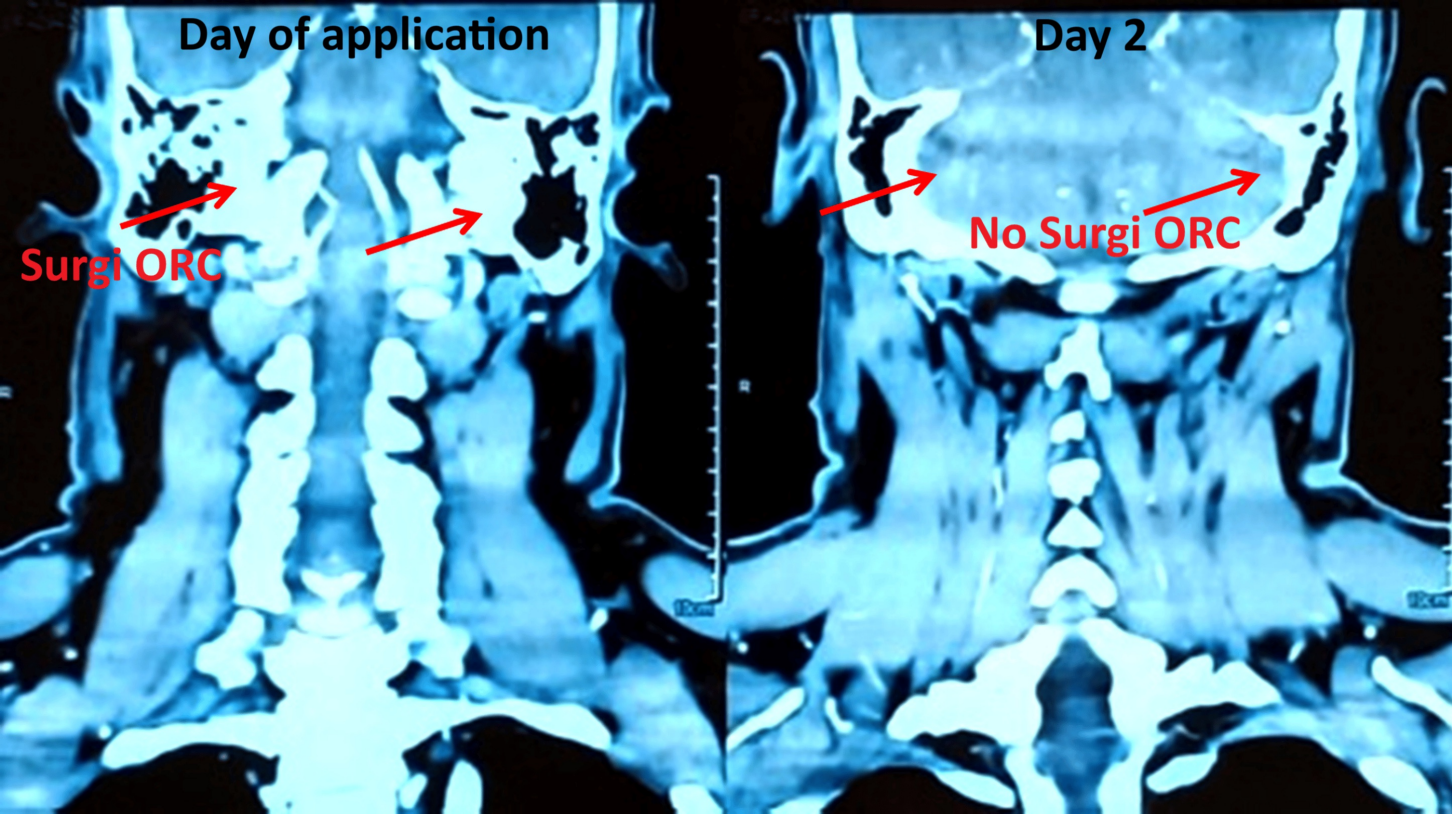
CT scan images for case 1 Left panel (day of application): Surgi-ORC® material (indicated by arrows) is visible in the operated sinus cavity immediately after application. Right panel (day 2): the same region shows no visible Surgi-ORC® material, indicating its absorption/degradation within two days.

A 52-year-old male presented with a longstanding right-sided parotid swelling that had been increasing in size for six to seven years. The swelling was non-tender and associated with mild discomfort during mastication. His medical history was nonsignificant, with no known comorbid conditions or family medical history. Physical examination revealed firm, non-tender swelling in the right parotid region. Facial nerve function was impacted. CT scan of the neck suggested a benign neoplasm of the superficial lobe of the parotid gland. The patient was admitted for parotidectomy. Preoperative vital signs were typical: blood pressure 120/70 mmHg, respiratory rate 22 breaths per minute, pulse rate 78 beats per minute, and body temperature 36.8°C. Under general anesthesia, a 5×6 cm preauricular incision curving below the earlobe within a natural skin crease was made to preserve postoperative aesthetics. The dissection advanced through the superficial lobe, ensuring careful identification and preservation of the facial nerve and its branches. The entire afflicted glandular tissue was removed, guaranteeing pristine margins. During dissection, moderate bleeding (5-10 mL) was encountered from the surgical bed, with total intraoperative blood loss of 50-60 mL. To ensure prompt hemostasis, a 10.2×10.2 cm Surgi-ORC® Knit variant was applied directly to the bleeding surface, facilitating clot formation and achieving adequate hemostasis in 1.9 minutes. The patient experienced a non-complicated recovery, with no complications of hematoma, facial nerve dysfunction, or surgical site infection. As the patient was stable, he was discharged on day 3. During the two-week follow-up, the incision exhibited satisfactory healing, and the patient did not experience any functional impairment. This particular case illustrates the efficacious application of Surgi-ORC® Knit in parotid surgery, especially in addressing mild hemorrhage in areas with intricate neurovascular structures. Its fast action and promising safety profile make it a viable option in ENT surgery for bleeding management. Figure 1 shows the CT scan of the complete absorption of Surgi-ORC® Knit on day 2.

Case 2

**Figure 2:**
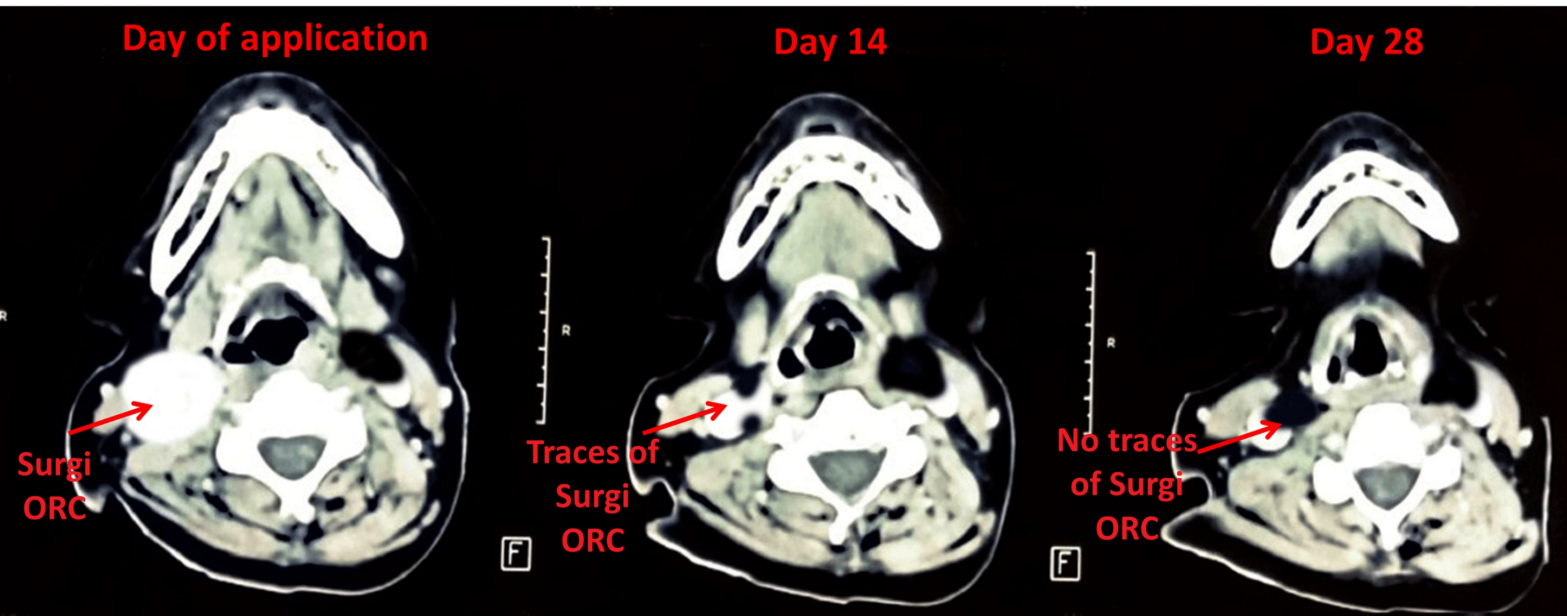
CT scan evaluation of Surgi-ORC® absorption over time for case 2 Day of application (left panel): Surgi-ORC® appears as a distinct hyperdense mass (arrow). Day 14 (middle panel): only traces of Surgi-ORC® are detectable at the application site. Day 28 (right panel): no visible traces of Surgi-ORC® remain, indicating complete absorption.

A 55-year-old Asian female presented with swelling on the left side of the thyroid gland associated with thyroiditis. The patient's medication and medical history revealed no significant findings. A contrast-enhanced computed tomography (CECT) scan of the neck (Figure 2) was performed to assess left thyromegaly with features of thyroiditis. Her vital signs were stable, with a temperature of 36.7°C, blood pressure of 100/80 mmHg, pulse rate of 76 bpm, and a respiratory rate of 22 breaths per minute. Under general anesthesia, a cervical incision of 2 cm was made above the sternal notch. The left thyroid lobe was identified and mobilized. During the procedure, moderate bleeding was observed from adjacent soft tissue and vascular structures, with a total blood loss of 60-70 mL. For effective bleeding control, 10.2×10.2 cm of Surgi-ORC® Knit was placed at the bleeding site, and within 2.05 minutes, hemostasis was achieved. On day 2 of follow-up, CT scan revealed the presence of Surgi-ORC® Knit, which warranted a follow-up CT scan on day 28, which showed complete absorption of Surgi-ORC® Knit (Figure 2). The patient exhibited satisfactory wound healing with no adverse events or complications till follow-up day 60. This case reinforces the clinical utility of Surgi-ORC® Knit as a viable option for managing bleeding in ENT surgeries involving inflamed and vascular tissue.

Case 3

**Figure 3:**
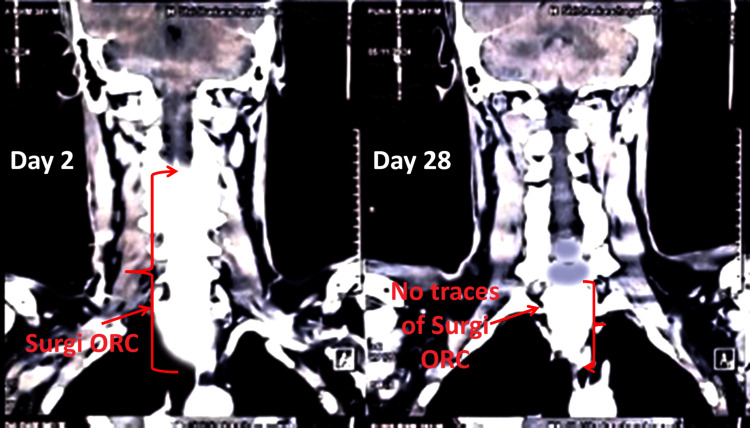
CT evaluation of Surgi-ORC® absorption for case 3 Day 2 (left panel): Surgi-ORC® is clearly visible at the application site (arrows). Day 28 (right panel): no visible traces of Surgi-ORC® remain, confirming complete absorption.

A 33-year-old Asian male was admitted to the hospital with a progressively enlarging mass in the right lateral region of the neck. The patient was experiencing mild dysphagia and discomfort during neck movement. There was no significant medical or medication history. On admission, his vital signs were stable: body temperature of 37.1°C, blood pressure of 110/80 mmHg, pulse rate of 78 beats per minute, and respiratory rate of 22 breaths per minute. As shown in Figure 3, CECT of the neck revealed a soft tissue lesion in the right lateral neck without definitive features of malignancy. Under general anesthesia, with a supraglottic airway (SGA) device used for ventilation, a right lateral neck incision was made to expose the affected area. Careful dissection was performed to isolate the lesion from adjacent soft tissue while preserving surrounding neurovascular structures. During the dissection, moderate intraoperative bleeding was encountered from vascularized soft tissues, with an estimated blood loss of 50-60 mL. Two sheets of Surgi-ORC® Knit, measuring 10.2×10.2 cm, were applied to the bleeding site to achieve hemostasis. Due to the extent of the bleeding, two ORC sheets were used, ensuring complete coverage. The knit structure of the hemostatic agent provided adequate adherence to the tissue, promoting rapid blood absorption and clot formation. The lesion was excised, and the wound was closed after confirming the absence of active bleeding. The patient remained hemodynamically stable throughout the procedure and was transferred to the postoperative recovery unit in satisfactory condition. Figure 3 shows CT images demonstrating the presence of Surgi-ORC® on day 2 and its complete absorption with no traces visible by day 28.

Case 4

**Figure 4:**
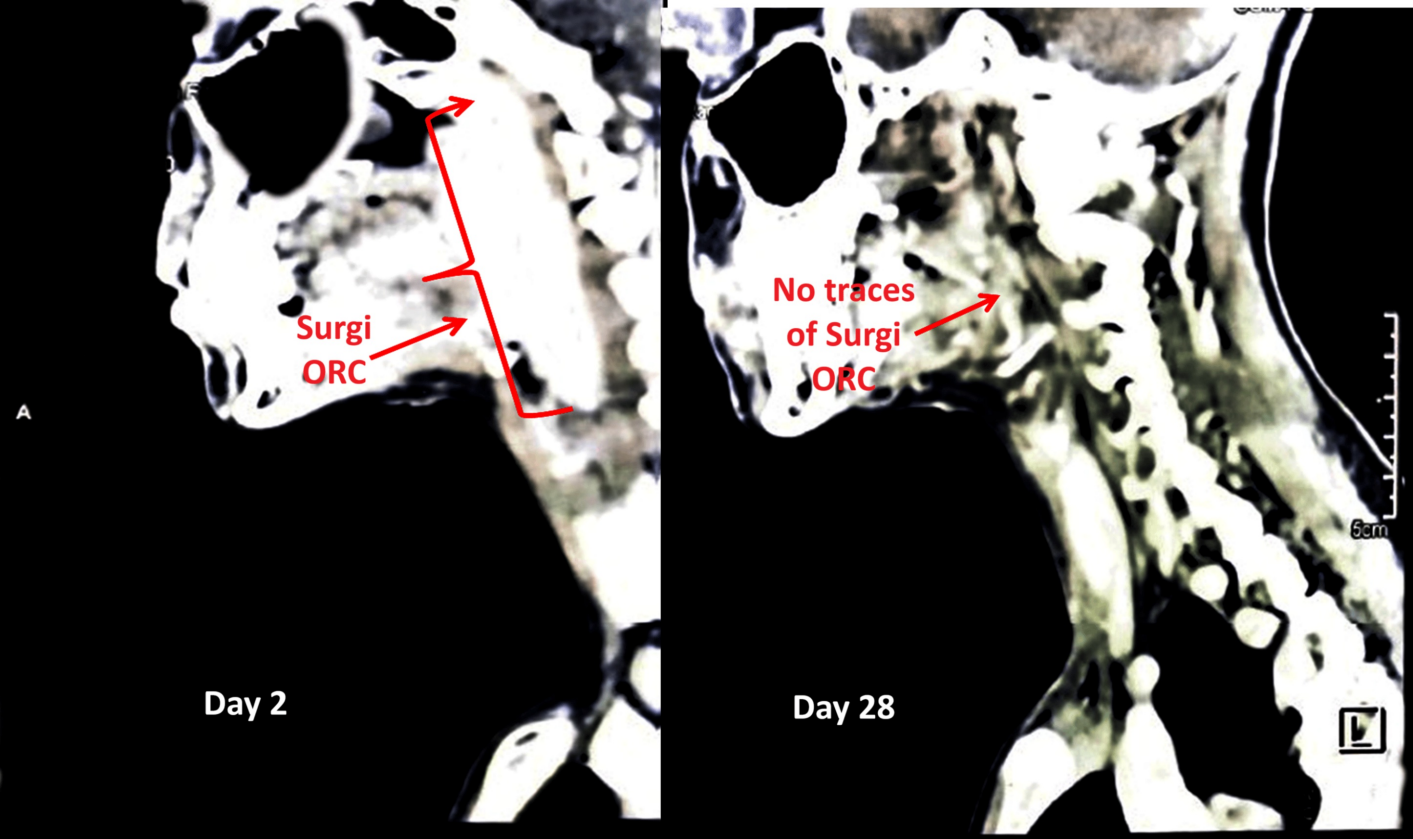
CT scan images of case 4 Day 2 (left panel): Surgi-ORC® is clearly visible in the operated region (arrows). Day 28 (right panel): no traces of Surgi-ORC® are observed, indicating complete resorption.

A 25-year-old Asian female presented to the hospital with swelling in the right submandibular region. Clinical examination revealed a firm, well-circumscribed mass beneath the mandible. Given the risk of recurrent infections, progressive enlargement, and potential obstruction of the salivary duct, surgical excision was deemed the most appropriate management strategy. Given the CECT results (Figure 4), the clinical team opted for surgical excision under general anaesthesia. The patient's medical and medication history were not significant. Her vital signs remained normal before surgery: body temperature of 36.8°C, blood pressure of 120/90 mmHg, pulse rate of 74 beats per minute, and respiratory rate of 20 breaths per minute. Surgery involved moderate bleeding with a total blood loss of 30-40 mL during surgery. A 5.1×10.2 cm-sized Surgi-ORC® Knit was positioned at the bleeding site to control bleeding (Figure 4). Following confirmation of adequate hemostasis, the surgical field was carefully examined for residual bleeding before concluding the procedure.

Clinical outcomes

This section presents outcomes of the four patients who underwent ENT surgery using Surgi-ORC® Knit for hemostasis. Patient characteristics are shown in Table 1. All patients encountered moderate bleeding during their respective surgical procedures. The mean intraoperative blood loss was 53 ± 9.79 mL (range: 30-70 mL), and the mean TTH was 1.75 ± 0.47 minutes (range: 0.93-2.1 minutes). These findings indicate consistent hemostatic performance of Surgi-ORC® Knit across all cases, regardless of the procedure type.

**Table 1:** Baseline characteristics of patients

Characteristics	Case 1	Case 2	Case 3	Case 4
Age	52	55	33	25
Gender	Male	Female	Male	Female
Presenting symptoms	Thyromegaly, discomfort	Neck pain, dysphagia	Lateral neck swelling	Swelling under the jaw
Procedure performed	Parotidectomy	Left thyroidectomy	Neck mass excision	Right submandibular gland excision
Intraoperative blood loss	50-60 mL	60-70 mL	50-60 mL	30-40 mL
Postoperative complication	None	None	None	None

Hemostatic efficacy

All patients encountered moderate bleeding during a surgical procedure, and the mean (SD) blood loss for all four patients was 52.5 (12.58) mL. TTH varied across all four patients. For parotidectomy (patient 1), hemostasis was achieved in 1.93 minutes, within the expected range of under 2 minutes. In left thyroidectomy (patient 2), hemostasis was reached in 2.05 minutes. The neck mass excision surgery (Patient 3) took 2.1 minutes to achieve hemostasis, remaining within the anticipated range of less than 5 minutes. Finally, the right submandibular gland swelling excision (patient 4) achieved hemostasis in just 0.93 minutes, within the expected threshold of under 2 minutes.

Safety and follow-up

A CT scan performed on day 2 revealed the absence of Surgi-ORC® Knit for a patient who was undergoing parotidectomy. However, for patients 2, 3, and 4, Surgi-ORC® Knit was visible on the CT scan performed on follow-up day 2. This warranted a follow-up CT scan on day 28, which revealed the absence of Surgi-ORC® Knit, supporting complete absorption. Detailed follow-up of all four patients is shown in Table 2.

**Table 2:** Follow-up outcomes of ENT surgeries using Surgi-ORC® Knit CECT, contrast-enhanced computed tomography; ORC, oxidized regenerated cellulose; TTH, time to hemostasis

Parameter	Case 1 (right-sided parotidectomy)	Case 2 (left thyromegaly with thyroiditis)	Case 3 (right lateral neck excision for supraglottic airway	Case 4 (right submandibular swelling excision)
Day 2 radiological examination (CECT scan of neck)	No ORC traces observed	ORC visualized	ORC visualized	ORC visualized
TTH	1.9 minutes	2.05 minutes	2.1 minutes	0.93 minutes
Wound healing progress	Healing noted; no re-bleeding and infection observed	Healing noted; no re-bleeding and infection observed	Healing noted; no re-bleeding and infection observed	Healing noted; no re-bleeding and infection observed
Dressing reinforcement needed	No	No	No	No
Day 28 radiological examination	Not performed; ORC presumed fully absorbed	Performed; no ORC traces observed, ORC presumed fully absorbed		
Day 60	Satisfactory healing	Satisfactory healing	Satisfactory healing	Satisfactory healing
Adverse events on day 2, day 28, and day 60	None	None	None	None

User feedback from the physician

**Figure 5:**
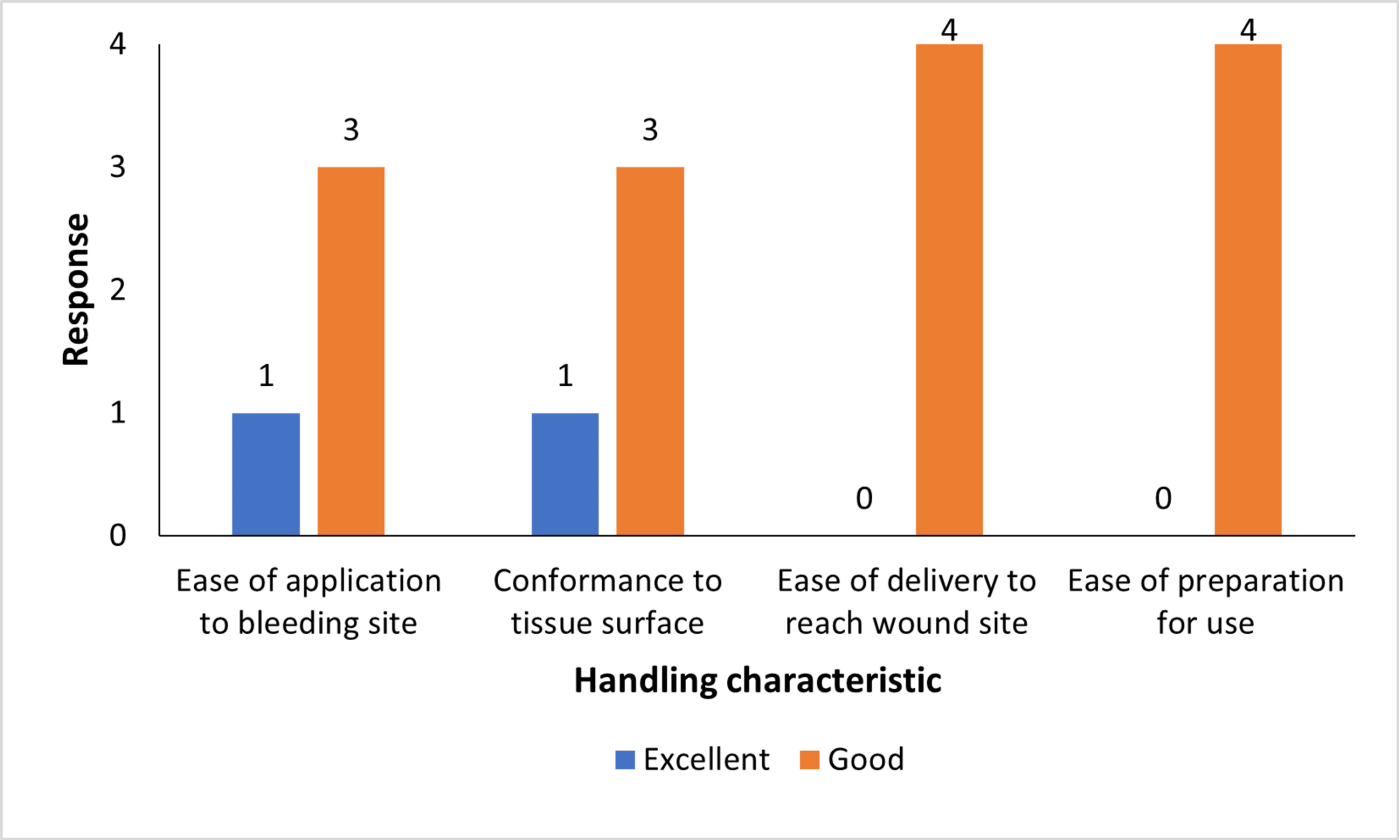
Surgeons' evaluation of the performance of the haemostatic product (Surgi-ORC®)

The product was developed with a strong focus on user-friendliness, incorporating clear and detailed handling instructions on the label to facilitate healthcare professionals' application. Its intuitive design ensures ease of use and high adaptability, minimizing the likelihood of application errors and enhancing operational efficiency during surgical procedures. Based on the evaluation, the surgeon rated the Surgi-ORC® Knit as "good" or "excellent" for the parameters investigated (Figure 5).

## Discussion

This case series aimed to evaluate the safety and efficacy of Surgi-ORC® Knit as a hemostatic agent in various ENT surgeries. The significant findings from our study are as follows: rapid hemostasis was achieved in less than 5 minutes, and complete biodegradation of Surgi-ORC® Knit was confirmed by the surgeon on postoperative day 28. TTH for Surgi-ORC® Knit ranged from 0.93 to 2.1 minutes, depending on the intricacy of the procedure. For parotidectomy, hemostasis was achieved in 1.93 minutes. On follow-up day 28, Surgi-ORC® Knit was absorbed entirely, and thus it is less likely to cause granuloma and development of foreign body reaction, as reported with other types of ORCs [9], which supports the safety profile of Surgi-ORC®. For thyroidectomy (patient 2), it took 2.05 minutes to achieve hemostasis, while lateral neck mass excision surgery took 2.1 minutes. TTH was the lowest for submandibular mass excision (patient 4), 0.93 minutes.

Subsequent assessments provided more insight into the efficacy of Surgi-ORC®. A CECT scan conducted on day 2 revealed no evidence of the product in the parotidectomy case. Residual ORC was identified in the remaining instances: left thyromegaly with thyroiditis, right lateral neck excision for SGA, and excision of right submandibular swelling. By day 28, the complete absorption of Surgi-ORC® Knit was verified in these instances; however, the patient undergoing parotidectomy was considered to have achieved complete absorption based on the day 2 scan. These findings correspond with the anticipated resorption timeframe of Surgi-ORC® within 28 days. At the 60-day follow-up, all patients demonstrated adequate wound healing, with no adverse events, including infections, adhesions, granuloma formation, or foreign body reactions, affirming the product's safety and efficacy. The safety profile of the Surgi-ORC®, with no adverse events recorded in this case series, aligns with the literature that supports the biocompatibility and safety of ORC products [10]. Additionally, low pH of ORC reduces the risk of prevalent post-surgical infection [11]. With moderate to low bleeding risk involved with ENT surgery, Surgi-ORC® Knit provides a flexible approach to control bleeding where conventional bleeding control methods may be ineffective [12]. Surgeons reported the hemostatic handling characteristics of Surgi-ORC® Knit as "good" to "excellent" in terms of application ease, adherence to tissue, and efficient delivery to the wound site.

It is important to acknowledge that all cases in this series involved moderate intraoperative bleeding (30-70 mL) and did not represent high-risk surgical scenarios. Traditional techniques such as electrocautery and nasal packing were available during these procedures but were not required, as hemostasis was achieved using Surgi-ORC®. Although no adverse events were observed in this case series, previous studies have reported potential complications associated with ORC, including granuloma formation and interference with postoperative imaging. Surgi-ORC® is designed for faster absorption and ease of handling, and our findings demonstrate its feasibility and satisfactory handling properties in selected ENT procedures. The absence of failed interventions reflects the moderate bleeding severity of the selected cases and should be considered when interpreting the findings. Consequently, these results provide exploratory evidence and should not be generalized to high-risk ENT procedures. Further research with larger cohorts, higher bleeding risk surgeries, and comparator arms is necessary to validate these findings and establish broader external validity.

This case series offers insights into the real-world use of Surgi-ORC® and its possible benefits in ENT surgery. The findings add to the increasing clinical evidence backing the use of Surgi-ORC® in otolaryngological procedures. Its noted effectiveness and safety indicate that it could be a helpful option for handling intraoperative bleeding and may improve the efficiency of surgical procedures.

## Conclusions

The findings of this case series suggest that Surgi-ORC® is a feasible and safe option for managing bleeding in ENT surgeries, achieving rapid hemostasis and complete biodegradation without observed adverse outcomes. Surgeons reported favorable handling characteristics. However, given the limited sample size, moderate bleeding severity, and absence of a comparator, these results should be interpreted as preliminary. Larger, controlled studies involving higher-risk procedures are warranted to validate the safety, efficacy, and broader clinical utility of Surgi-ORC® in ENT surgery.
